# Paraneoplastic Cough and Renal Cell Carcinoma

**DOI:** 10.1155/2016/5938536

**Published:** 2016-03-21

**Authors:** Stephen Sullivan

**Affiliations:** University of Victoria, 703-240 Douglas Street, Victoria, BC, Canada V8V 2P3

## Abstract

A case of patient with intractable cough due to renal cell carcinoma is reported. The discussion reviews the literature regarding this unusual paraneoplastic manifestation of renal malignancy.

## 1. Case Presentation

A 62-year-old woman with a history of mild seasonal asthma and gastroesophageal reflux presented with an eight-month history of cough. Initially it was “… very much like an asthma cough. Very little phlegm and just aggravating.” Eventually it became intractable occurring “any time during the day or night, except when I was sleeping.” The cough was worse with exertion, cold air, and talking. It caused posttussive vomiting. “I would be wracked with uncontrolled coughing that would leave me hot, weak, sweating, exhausted and usually having vomited.” In the few months prior to presentation she had also experienced profound fatigue, drenching night sweats, and a 25–30-pound weight loss but no documented fever (quotation marks indicate the patient's words).

Physical examination was normal with no pulmonary wheezes or crackles, no abdominal masses, and no lymphadenopathy.

Inhaled corticosteroids, bronchodilators, oral prednisone, montelukast, and esomeprazole did not help. Oral hydrocodone gave temporary relief.

Fortuitously a high resolution CT scan of the chest included her upper abdomen and revealed a 6.5 cm heterogeneous lesion in the upper pole of the right kidney with local lymphadenopathy but no involvement of the diaphragm. There were a lytic lesion in the body of L4 and three small pulmonary nodules, the largest being 1.7 cm.

Also fortuitously the patient's presentation to our emergency room coincided with the publication the same week of a very similar case in the Canadian Medical Association Journal [1].

A laparoscopic right radical nephrectomy and lymph node dissection were performed. The diaphragm was not involved. The tumor was a clear cell carcinoma with sarcomatoid differentiation. The renal vein and one of seven nodes were involved. Postoperatively the patient stated, “My cough seemed to disappear with my kidney. I was glad to wish both a farewell.” Her L4 lesion was irradiated and she was treated with sunitinib. Unfortunately she developed widespread bony metastases and died 14 months after the start of her cough. During the metastatic course of her illness the cough did not return but she was on large doses of narcotics for bony pain.

## 2. Discussion

Renal cell carcinoma (RCC) has been called “the internist's tumor” [2]. Its paraneoplastic manifestations are myriad as follows.

Some of the paraneoplastic manifestations of RCC are

 fever;
 sweats;
 weight loss;
 fatigue;
 anemia;
 erythrocytosis;
 neutrophilia;
 eosinophilia;
 thrombocytosis;
 hypercalcemia;
 gynecomastia or galactorrhea;
 hyper- and hypoglycemia;
 hypertension;
 cushing syndrome;
 amyloidosis;
 hepatic dysfunction;
 nephropathy;
 peripheral neuropathy;
 motor neuron disease;
 cerebellar ataxia;
 opsoclonus-myoclonus;
 myasthenia gravis;
 limbic encephalitis;
 stiff person syndrome;
 esophageal dysmotility;
 polymyalgia rheumatica;
 polymyositis;
 mania.

The classic clinical triad of loin pain, flank mass, and hematuria pale in comparison.

Cough due to RCC has been noted in the English literature for over 80 years [3]. It was thought to be due to pulmonary metastases or diaphragmatic irritation [4]. However a review in 1960 referenced a Swedish publication [5] of “… an interesting, but totally unexplained, finding… namely a severe cough which disappears after nephrectomy” [6]. Another review in 1963 mentioned a patient without metastases whose primary symptom of “paroxysmal cough” resolved after nephrectomy [7].

I have found reports of 11 patients (5 men, 6 women; age 24–68 years) with RCC whose cough appeared to be paraneoplastic rather than metastatic [1, 5, 8–14]. The oldest was published in Spanish in 1943 and aptly titled,* Tos Renal, su importancia clinica*, “Renal Cough, Its Clinical Importance” [8].

From these reports I believe a recognizable pattern emerges.

The cough has been described as “dry, hacking, nonproductive, severe, constant, persistent, refractory, recalcitrant, intense, intolerable, intractable and obstinate.” One report even called it “the cough from hell” [14]. It may interfere with the ability to sleep, eat, drink, and even speak. Posttussive vomiting is mentioned in two cases.

The reports suggest that the cough responds poorly to standard antitussives; however narcotics, intravenous corticosteroids, and oral diazepam may be worth trying [13, 14]. In my patient's case hydrocodone seemed to help.

The cough may precede the eventual diagnosis of RCC by up to 24 months and be accompanied by fever, sweats, weight loss, and a high ESR. In all cases the cough resolved promptly, even “upon awakening from surgery” [11], when the tumor was resected, debulked, embolized, or irradiated. In two cases cough returned with the tumor recurrence but resolved when the metastases were resected [11, 13].

Although cough with RCC may be due to pulmonary metastases or diaphragmatic irritation, these reports suggest that it may also be due to a humoral substance produced by the tumor. What is the substance? It is not known. Prostaglandins, interleukin-6, and bradykinin have been suggested [2, 12, 13]. Indeed the pattern of my patient's cough leads me to search her pharmacy record for an ACE inhibitor.

However, paraneoplastic or metastatic, in the end it matters not. Only that the inquiring physician considers RCC as a possible cause of the patient's intractable cough and searches for a treatable lesion.

## Learning Objectives

(i) Recognize cough as a paraneoplastic presentation of renal cell carcinoma.
(ii) Be aware of the American College of Chest Physicians 2014 overview of the management of cough [15].

## Pretest

(i) What conditions should be considered in a patient with or without a normal chest X-ray and a chronic cough?
(ii) What minor modification in the investigation of chronic cough might lead to an earlier diagnosis of renal cell carcinoma as its cause?

## Posttest

- What conditions should be considered in a patient with or without a normal chest X-ray and a chronic cough?
- The latest American College of Chest Physicians overview on cough lists 17 potential causes of chronic cough. The top five are upper airway cough syndrome (formerly post nasal drip), asthma, nonasthmatic eosinophilic bronchitis, gastroesophageal reflux disease, and COPD. Number 16 is “uncommon causes.”
- What minor modification in the investigation of chronic cough might lead to an earlier diagnosis of renal cell carcinoma as its cause?
- Including the upper abdomen in a high resolution CT scan of the chest should identify a RCC. It did in our patient.
